# Complete Genome Sequence of the Circular Extrachromosomal Element of *Naegleria gruberi* Strain EGB Ribosomal DNA

**DOI:** 10.1128/genomeA.00020-18

**Published:** 2018-02-08

**Authors:** John C. Mullican, Nora M. Chapman, Steven Tracy

**Affiliations:** a Department of Pathology and Microbiology, University of Nebraska Medical Center, Omaha, Nebraska, USA

## Abstract

The circular extrachromosomal element of *Naegleria gruberi* strain EGB was linearized, molecularly cloned, and fully sequenced. The sequence comprises 14,007 bp and encodes the organism’s rRNA genes, two potential open reading frames, and numerous repeated sequence regions.

## GENOME ANNOUNCEMENT

Amoebae of the *Naegleria* genus (family *Vahlkampfiidae*) encode a single copy each of the 5.8S, 18S, and 28S rRNA genes on 3,000 to 5,000 copies of an extrachromosomal element (1, 2); no rRNA genes are in the nuclear DNA (3). This extrachromosomal ribosomal DNA (rDNA) location, first shown in *Naegleria gruberi* (1), also exists for other *Naegleria* species as well as *Entamoeba histolytica* (4–6). The extrachromosomal element and the nuclear DNA of a different strain of *N. gruberi* were sequenced earlier (3, 7). We report here the sequence of the rDNA plasmid from a different *N. gruberi* strain (EGB).

Total *N. gruberi* DNA was cleaved with BstI to linearize the extrachromosomal element DNA. The band at 14 kbp was cut from agarose gels, and DNA was isolated and ligated into the BstI-linearized pGEM7Zf(+) vector (Promega Corp.). Clones with inserts of 14 kbp were restriction mapped, and subclones were used for sequence analysis. Sequencing was performed in both directions by using T7 DNA polymerase (Sequenase v. 2.0) (ThermoFisher Scientific). G+C-rich regions were sequenced utilizing the dideoxynucleoside triphosphate (ddNTP) chain termination method (8). dITP was substituted for dGTP for the most straightforward approach. *Taq* DNA polymerase with 7-deaza-GTP (TaqTrack sequencing kit, Promega Corp.) was also used for difficult compressions. Sequence data were analyzed with the EuGene and Sequence Assembly Manager programs (Baylor College of Medicine). Sequences were aligned with the IBI AssemblyLIGN program (Eastman Chemical). Verification of some contig assemblies was carried out using assembly programs in the GCG sequence analysis software package (GCG, Madison, WI).

The *N. gruberi* EGB extrachromosomal element (pNgrubEGB) comprises 14,007 bp with an overall G+C content of 40.8%. Comparison of sequences that flank the BstI site used to clone the EGB plasmid reported here with those of the previously published NEG-M sequence (GenBank accession no. AB298288) (7) confirmed that the flanking sequences and the BstI site between the plasmids of the two strains are identical (7). The rDNA region is 5,855 bp (including two internal transcribed spaces [ITS]) with 46.5% G+C content, while the non-rDNA sequence (NRS) is 8,152 bp with 36.7% G+C content. The NRS contains 7 repetitive DNA sequences (expected threshold at 10) accounting for 2,526 bp and approximately 18% of the total episomal DNA. The repeats range in size from 34 bp to 371 bp. The NRS alone is 31% repetitive DNA. The episome contains 18% repetitive DNA, 3.5-fold more than the 5.1% in the genome (3). Sequence analyses of rDNA plasmids from other *Naegleria* spp. should aid in identifying conserved repetitive sequences or potential higher-order structures. Two yeast autonomously replicating sequence (ARS) consensus sequences were identified in the NRS region in opposite orientation to the rDNA (9). Two putative open reading frames (ORFs) were annotated. One, likely a pseudogene, was located downstream of the 28S rRNA gene and showed limited similarity to *Naegleria*-homing endonucleases (10). The second ORF was upstream of the 18S rRNA gene; translation did not result in a protein with similarity to published sequences.

### Accession number(s).

The complete sequence has been deposited in GenBank under the accession no. MG699123. The version described in this paper represents the first version, MG699123.1.
